# Anesthesia for ERCP: Impact of Anesthesiologist's Experience on Outcome and Cost

**DOI:** 10.1155/2013/570518

**Published:** 2013-05-28

**Authors:** Basavana G. Goudra, Preet Mohinder Singh, Ashish C. Sinha

**Affiliations:** ^1^Department of Anesthesiology and Critical Care Medicine, Hospital of the University of Pennsylvania, 3400 Spruce Strett, 680 Dulles, Philadelphia, PA 19104, USA; ^2^Department of Anesthesia, All India Institute of Medical Sciences, New Delhi, India; ^3^Department of Anaesthesia, Drexel University College of Medicine, University of Pennsylvania, Philadelphia, PA, USA

## Abstract

The present study evaluates the effect of anesthesiologist's experience in providing deep sedation for endoscopic retrograde cholangiopancreatography (ERCP) on cost and safety. *Methodology*. Perioperative records of 1167 patients who underwent ERCP were divided on the basis of anesthesiologist assisting these procedures either on regular basis (Group R) or on ad hoc basis (Group N). Comparisons were made for anesthesia times, complication rates, and airway interventions. *Results*. Across all American Society of Anesthesiologists (ASA) Classes, regular anesthesiologists were more efficient (overall mean anesthesia time in Group R was 24.82 ± 12.96 versus 48.63 ± 21.53 minutes in Group N). Within Group R, anesthesia times across all ASA classes were comparable. In Group N, anesthesia times for higher ASA status patients were significantly longer (ASA IV, 64.62 ± 35.78 versus ASA I, 45.88 ± 11.19 minutes). Intubation rates (0.76% versus 12.8%) and median minimal oxygen saturation (100% versus 97.01%) were significantly higher in Group R. Had Group R anesthesiologists performed all procedures, the hospital could have saved US $ 758536 (based upon operating room time costs). *Conclusion*. Experience in providing deep sedation improved patient safety and decreased the operating room turnaround time, thereby lowering operating room costs associated with these procedures.

## 1. Introduction 

Achieving “efficiency without compromising safety” is the new mantra in medicine. Specialization and training in a chosen area have already been implemented successfully in many fields of anesthesiology. Many areas like cardiac, obstetrics, pediatric, and neuroanaesthesia have already received recognition with dedicated fellowships programs. However, anesthesia for many procedures that are done by anesthesiologists in remote diagnostic/therapeutic locations (out of operating room (OR)) is poorly studied. Most of these procedures are conducted under deep sedation (previously termed “Monitored Anesthesia Care (MAC)”). The risk of complications in out of OR is similar to OR anesthesia [1]. Although formal specialization may not be necessary in this growing field, having a core group of interested anesthesiologists might help to drive efficiency without altering the safety. The continuum from conscious sedation to deep sedation and sometimes to general anesthesia is more likely to be recognized and managed better by anesthesiologists regularly involved in such care, thus reducing complication rates [2].

 The effect of anaesthesiologist's experience (in the setting of ERCP) on the safety and cost of providing deep sedation has not been studied. In the current analysis, we have tried to address this aspect of anesthesia care in patients undergoing ERCP with anesthesia assistance. 

## 2. Methods

After institutional review board approval, perioperative records of patients who underwent ERCP at the Hospital of the University of Pennsylvania from 2010 to 2011 were analyzed. In this retrospective exploratory study, the following patient-specific and procedure-specific data were extracted: demographic profile and comorbidities (ASA class), airway evaluation, (Modified Mallampati class) MMP, indication for ERCP, method of airway management, minimal oxygen saturation during the procedure, persistent cardiovascular complications if any, duration of ERCP, total duration of procedure, and any procedure cancellation after the start of the procedure due to anesthesia issues. Total duration of procedure was defined as the time from the patient entering the gastroenterology (GE) suite to leaving the suite. Duration of ERCP was defined as the time interval between endoscopy probe insertion to its removal. For all patients anesthesia time (for this study) was calculated as the time difference between “total duration” and “duration of ERCP.” Hospital of the University of Pennsylvania has two gastroenterology suites, one attached to the main hospital block and the other attached to outpatient block (Perelman center for advanced medicine, surgicenter). The procedures done in surgicenter are supervised by a small group of anesthesiologists with interest and extensive experience in providing deep sedation. This group of anesthesiologists involved in Surgicenter primarily involved with GE procedures was labeled as “regular” (R) for the study purpose. The GE suite attached to the main hospital is supervised by anesthesiologists primarily involved in general anesthesia in the main operating room complex and are assigned to GE suite only on an ad hoc basis. This group of anesthesiologists was labeled as “nonregular” (N). GE suites at both of these locations are equivalent in terms of available monitoring, resuscitation equipment, and anesthesia delivery systems. The use of the term “surgicenter” in our setting might be misleading; hence, an explanation is necessary. Unlike most surgicenters in the country, we do not have any restrictions on the ASA class of the patients or procedural difficulty. It is not uncommon to provide anesthesia care for patients with severe aortic stenosis, pulmonary hypertension, severe (chronic obstructive airway disease COPD), pacemaker/defibrillator, cardiomyopathy evaluated for cardiac transplant, and so forth. Because the backup help available (including ICU admission) is similar in both facilities, we do not make any distinction, and this is the hospital's stated policy. However, the surgicenter patients are cared by a small group of anesthesiologists. This allowed us to make comparison of naturally segregated groups, yet comparable populations. Comparisons were made for complications, airway methods, and anesthesia times and estimated operating room costs attributable to anesthesia times. 

## 3. Statistical Analysis

The statistical analysis was done using SPSS Version 20 (IBM Inc., Chicago, IL, USA) for Macintosh. The level of statistical significance was set to allow an alpha error of 5% (*P* value of 0.05). Descriptive statistics were used to define distribution of parameters among the groups. Baseline nonparametric, ordinal data among the groups were compared using Mann-Whitney's test. Equality of variances of parametric variables was tested using Levene's test for equality. One-way ANOVA was used to compare the mean anesthesia times of ASA subgroups within both Group R and Group N. Post hoc analysis was done using Tukey's HSD test. Student's unpaired *t*-test was used to compare means of parametric data between the groups. Pearson's Chi-square test was used to relate airway method frequency data to specifically ASA grade (IV) and also with MMP class IV patients. Automatic linear regression modeling of SPSS was used to evaluate the relative effect of combined factors: anaesthesiologist group, ASA status, airway intervention, and MMP on variations in anesthesia time. 

## 4. Results

A total of 1167 ERCP procedures were performed, of which 653 (56%) were assisted by regular (Group R) and 514 (44%) were assisted by nonregular (Group N) anesthesiologists. Group R had 233 (35.68%) females and 420 (64.31%) males, whereas Group N had 207 (40.27%) females and 307 (59.72%) males. The mean age of patients in both groups was statistically comparable (58.11 ± 16.33 and 60.55 ± 14.18 in Group N and Group R, resp.). The overall observed mean BMI (kg/m^2^) was 27.56 ± 7.01 (27.93 ± 6.95 and 27.20 ± 7.04 in Group N and Group R, resp.). Mean BMI values in both groups were found to be statistically equivalent (*P* = 0.07) using unpaired student's *t*-test. In Groups N and R 79.57% (376 +33) and 56.35% (361 + 7) were of ASA class III and IV (combined), respectively. The MMP distribution between both groups was compared using Wilcoxon rank-sum test and was found to be similar in both groups (*P* = 0.714).

Mean anesthesia time was found to be significantly higher in Group N using Student's *t*-test (*P* < 0.001). Anesthesia time in Group N (48.63 ± 21.53) was almost twice that of Group R (24.83 ± 12.96). Mean anesthesia times of individual ASA grades within each group were compared using one-way ANOVA. Within Group N, highly significant difference was found between anesthesia times of ASA subgroups (*P* < 0.001), whereas the same was not significant in Group R (*P* = 0.518). On post hoc analysis, all ASA groups had statistically similar anesthesia times in Group R, whereas in Group N ASA III/IV had significantly higher time than ASA I/II. (*P* < 0.05) (Table 1) Unpaired *t*-test on comparison for anesthesia times between corresponding ASA subgroups of both Group R and Group N showed significantly higher duration in all ASA subpopulations of Group N (Table 1).

**Figure 1:**
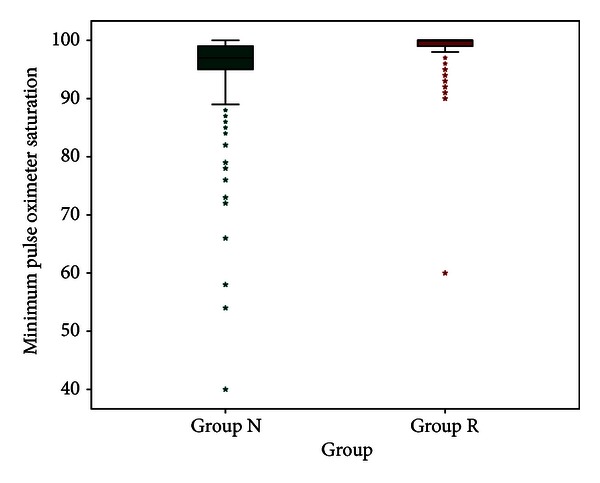
Box-and-whiskers graph showing distribution of minimal oxygen saturation (whiskers show 95 percentile range) in both groups. The stars represent outliers in both groups.

**Figure 2:**
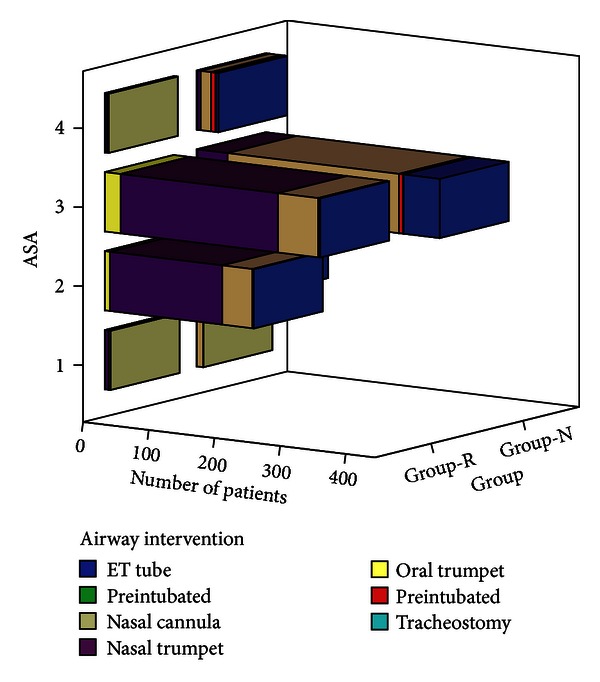
Graph showing number of patients with ASA distribution, airway intervention in both groups.

Minimum documented pulse oximeter saturation values were compared using Man-Whitney's test and were significantly lower in Group N (*P* < 0.001). The median minimal saturation in Group R was 100% (60–100%) and in Group N was 97.01% (40–100%) (Figure 1). All these desaturation episodes were transient and did not require any active airway intervention. ASA distribution between groups showed significant difference using Mann-Whitney's test (*P* < 0.001), with higher number of ASA IV and III patients in Group N. Distribution of MMP score was similar between both groups (*P* = 0.8). The airway interventions in both the groups are represented in (Table 2), (Figure 2). In view of infrequent need for endotracheal intubation, the reasons are either documented or could be inferred. Five patients were intubated for procedure by Group R in anticipation of increased risk of aspiration due to history of gastroesophageal reflux disease. Sixty-six patients were intubated prior to procedure by Group N where commonest indications were inferred to be preoperative snoring/obstructive sleep apnea, increased BMI, and elderly age group. However, Pearson's Chi-square test evaluating frequency of intubation with increasing difficult airway (higher MMP-IV) or sicker patients (ASA IV) showed no correlation between the two (*P* = 0.992).

 SPSS derived automated linear regression modeling was used to evaluate the predictability of anesthesia time for regular versus nonregular anesthesiologists. Factors like airway assessment (MMP), ASA status, and type of airway device used could predict anesthesia duration with only 41.6% accuracy. Among these, anesthesiologist's skill (regular versus nonregular) accounted for 50% of predictability, type of airway intervention accounted for 47%, ASA status 3% (*P* < 0.05  for above three) and MMP 1% (*P* > 0.05).

We calculated the cost based on an average operating room cost in USA [3] of $ 62 per minute of the procedure time. This does not include the cost of equipment required for the ERCP. In Group R, the mean cost on total time basis was 3402.99 ± 1506.58$, of which anesthesia time cost accounted for 45.2% of the total (1539.42 ± 803.53$). Similarly in Group N, the mean cost on the total time basis was 5069.05 ± 2379.78$, of which anesthesia time cost was (59.47%) 3015.17 ± 1336.26$. The mean difference of total time based cost and anesthesia time attributable cost among the groups was 2206.06 ± 118.942$ (*P* < 0.001) and 1475.75 ± 71.346$ (*P* < 0.001), respectively. The mean ERCP procedural duration was higher in Group N (29.77 ± 1.44 minutes) by 10.61 ± 1.55 minutes than in Group R (19.08 ± 0.80 minutes), this accounted for $ 657.82 ± 96.10 (29.76%) of the mean total cost difference. However, anesthesia time alone amounted for the remaining 70.24% of mean total cost differences. The anesthesia time showed poor correlation with ERCP procedure time. (Pearson's correlation coefficient “*r*” = 0.213, *P* < 0.001). 

## 5. Discussion

In the current analysis, we have compared the performance of two groups of anesthesiologists providing deep sedation to similar patient groups in two different areas. Although it is a retrospective review, the strength of results lies in the fact that comparisons could be made between similar groups. The results highlight the time-based efficiency of the anesthesiologists providing deep sedation on a regular basis along with increased safety and a possible economic advantage. This is supported by significantly lower anesthesia times and higher values of minimal oxygen saturation noted during the procedures supervised by “regular anesthesiologists.” Our analysis shows that had the procedures in Group N been performed by Group R anesthesiologists, it could have saved $ “758536 ± 36668” in a single center, along with increased patient safety. Extrapolating these figures to an estimated half a million ERCPs done yearly in the USA, [4] it would amount to an annual health sector saving of about US $ 740 million. Ours is a major hospital in the country performing large number of these procedures; the anesthesia times and procedure times are likely to be even longer in other hospitals. These findings become all more relevant in view of the mandate by both American Society of Anesthesiologists (ASA) and Royal College of Anesthetists (UK), for a propofol-based sedation to be administered by certified anesthesia providers only [2, 5, 6]. However, in most centers around the world, sedation for ERCP is still administered by gastroenterologists performing these procedures [7, 8]. Anesthesia provider's service is available for these procedures only on ad hoc basis [9]; thus, the situation in terms of anesthesia experience is very similar or even more limited than the present Group N. 

 The Group R anesthesiologists in this analysis had been involved in procedures performed under deep sedation on a regular basis. Most had been anesthesia consultants for around 10 years and had spent an average of 20–40% of their clinical time in GI endoscopy suite. Increased experience lead to comparable anesthesia times irrespective of associated co-morbidities/ASA status as suggested by statistically similar anesthesia times for patients cared by Group R (19.35 versus 25.39 minutes for ASA I versus IV, resp.). Higher number of ASA III/IV patients in group N was expected to increase anesthesia times; however, linear regression model evaluating the predictability of ASA status on anesthesia time (whether sicker patients required more time) showed poor regression coefficient and thus negated the same. 

On an average, anesthesiologists infrequently involved with procedures conducted with deep sedation (Group N) took almost 1.8 times more anesthesia time across all patients. This increased time was not only higher in sicker patients (ASA IV) but also proportionately larger in relatively healthier patients (ASA I) as well. Within Group N, increasing level of patient's sickness (ASA I to ASA IV) increased the anesthesia time by a factor of 1.6, while such a significant increase was not noted for ASA IV patients of Group R (Table 1). In spite of increased time (or because of), overall desaturation rates were higher in Group N. These findings suggest that “deep sedation-” based specialized anesthesia training is likely to decrease anesthesia turnaround time in all category of patients.

 The debate “to intubate or not to intubate” patients presenting for ERCP under deep sedation continues. In the current study, the indications for endotracheal intubation were not clear in Group N. Most of these indications (as documented in patient's anesthesia charts) were well-known risk factors for airway difficulties under general anesthesia (increasing BMI, history of OSA, and elderly). Due to semiprone positioning, the possibility of upper airway obstruction due to tongue fall with sedation is low [10]. Anesthesiologists with higher experience in performing deep sedation for ERCP might recognize the same; however, nonregular anesthesiologists (with primary experience in general anesthesia) are more likely to follow practices that hold true for general anesthesia and intubate these patients. This is consistent with the finding of an endotracheal intubation rate of 0.76% (5/653) in Group R versus 12.8% (66/514) in Group N. An almost 17 times higher endotracheal intubation rate in Group N cannot be explained by higher morbidities (compared to Group R) alone, as their intubation rate of sickest patient ASA IV (12.1%) was in fact lower than that of ASA III (14.7%) patients. Group N also intubated 7.3% of ASA II patients, in contrast to group R where no ASA IV patient was intubated and much lower intubation rates were seen in ASA III (0.8%) and ASA II (0.8%) subgroups. This probably highlights that level of experience favorably affects anesthesiologist's expertise in maintaining patent natural airway under deep sedation. 

Unlike procedures conducted under general anesthesia, where perioperative complications strongly relate to preoperative ASA status, ERCP in multiple other trials have shown no significant relation among the two [11, 12]. Our findings are in agreement with those of Amornyotin et al., who concluded that complications rates during ERCP were primarily dependent upon the skill of anesthesiologist rather than ASA status in elderly [12]. 

Commonest complications of deep sedation are respiratory depression (leading to desaturation) and hypotension. ERCP-based studies show that these are primarily transient, reversible and resolve with simple airway interventions or vasopressor boluses, leaving no sequelae [11]. None of the patients in either groups suffered persistent hypotension, and all such episodes responded to boluses of vasopressors. Transient desaturation episodes (responding to oxygen supplementation or decreasing sedation dose) were seen in both groups. Group R patients had lower number of episodic desaturation with significantly higher percentile of patients in higher saturation group (Figure 1). These episodes, as previously reported by Amornyotin et al., can have a possible bearing to improve anesthesiologist's skill acquired with increasing experience [12]. 

The present analysis has limitations of being a retrospective review. The documentation is generally done with no plans of analysis at a later date. Details for few parameters in some patient's anesthesia charts were missing, thus bringing down the actual sample size pertinent to that parameter. It would have been interesting to divide anesthesia times into “induction” and “recovery” phases to further analyze where the level of experience helps, but due to limitations in recording methodology, the same was not possible. We could not compare long-term complications rates as anesthesia charts used for analysis had records of immediate periprocedural period only. Hemodynamic variations like hypotensive episodes were also not compared as no significant persistent episode requiring specific attention (other than boluses of vasopressors) was recorded. The number of ASA IV patients in Group R was much smaller than that in Group N. Although this was incidental, it decreased the statistical significance of results. 

## Figures and Tables

**Table 1:** Comparison of anesthesia times between various ASA classes in both groups.

Mean anesthesia time						
Groups	Nonregular	%-Population	Regular	%-Population	Mean difference	Statistical significance
ASA I	45.88 ± 11.19	1.86%	19.63 ± 5.263	1.7%	26.25 ± 4.37	*P* < 0.001
ASA II	45.15 ± 15.68	18.41%	24.08 ± 10.83	39.14%	21.07 ± 1.93	*P* < 0.001
ASA III	48.62 ± 21.00	73.65%	25.33 ± 12.11	58.78%	23.53 ± 1.47	*P* < 0.001
ASA IV	64.62 ± 35.78	6.06%	25.39 ± 12.27	1.27%	39.28 ± 8.58	*P* = 0.014

**Table 2:** “Airway interventions” in both groups.

Airway interventions								
ASA status	Group	Endotracheal tube	Emergency intubation	Nasal cannula	Nasal trumpet	Oral trumpet	Preintubated	Tracheostomy
ASA I	Group R	0	0	3	7	0	0	0
	Group N	0	0	10	0	0	0	0

ASA II	Group R	2	0	46	171	8	0	0
	Group N	7	2	66	20	0	0	0

ASA III	Group R	3	0	61	240	24	0	0
	Group N	55	1	261	46	0	6	1

ASA IV	Group R	0	0	2	5	0	0	0
	Group N	4	0	18	7	0	7	1
